# M1 macrophage-derived exosomes inhibit cardiomyocyte proliferation through delivering miR-155

**DOI:** 10.1186/s12872-024-03893-0

**Published:** 2024-07-16

**Authors:** Xiaoqing He, Shan Liu, Zhanyu Zhang, Qirui Liu, Juan Dong, Zhifeng Lin, Junhao Chen, Lihuan Li, Weihua Liu, Shaojun Liu, Shiming Liu

**Affiliations:** Department of Cardiology, Guangdong Key Laboratory of Vascular Diseases, The Second Affiliated Hospital, Guangzhou Institute of Cardiovascular Disease, Guangzhou Medical University, Guangzhou, 510260 People’s Republic of China

**Keywords:** M1 macrophage, Exosomes, Mir-155, Myocardial infarction, Cardiomyocyte proliferation

## Abstract

**Background:**

M1 macrophages are closely associated with cardiac injury after myocardial infarction (MI). Increasing evidence shows that exosomes play a key role in pathophysiological regulation after MI, but the role of M1 macrophage-derived exosomes (M1-Exos) in myocardial regeneration remains unclear. In this study, we explored the impact of M1 macrophage-derived exosomes on cardiomyocytes regeneration in vitro and in vivo.

**Methods:**

M0 macrophages were induced to differentiate into M1 macrophages with GM-CSF (50 ng/mL) and IFN-γ (20 ng/mL). Then M1-Exos were isolated and co-incubated with cardiomyocytes. Cardiomyocyte proliferation was detected by pH3 or ki67 staining. Quantitative real-time PCR (qPCR) was used to test the level of miR-155 in macrophages, macrophage-derived exosomes and exosome-treated cardiomyocytes. MI model was constructed and LV-miR-155 was injected around the infarct area, the proliferation of cardiomyocytes was counted by pH3 or ki67 staining. The downstream gene and pathway of miR-155 were predicted and verified by dual-luciferase reporter gene assay, qPCR and immunoblotting analysis. IL-6 (50 ng/mL) was added to cardiomyocytes transfected with miR-155 mimics, and the proliferation of cardiomyocytes was calculated by immunofluorescence. The protein expressions of IL-6R, p-JAK2 and p-STAT3 were detected by Western blot.

**Results:**

The results showed that M1-Exos suppressed cardiomyocytes proliferation. Meanwhile, miR-155 was highly expressed in M1-Exos and transferred to cardiomyocytes. miR-155 inhibited the proliferation of cardiomyocytes and antagonized the pro-proliferation effect of interleukin 6 (IL-6). Furthermore, miR-155 targeted gene IL-6 receptor (IL-6R) and inhibited the Janus kinase 2(JAK)/Signal transducer and activator of transcription (STAT3) signaling pathway.

**Conclusion:**

M1-Exos inhibited cardiomyocyte proliferation by delivering miR-155 and inhibiting the IL-6R/JAK/STAT3 signaling pathway. This study provided new insight and potential treatment strategy for the regulation of myocardial regeneration and cardiac repair by macrophages.

**Supplementary Information:**

The online version contains supplementary material available at 10.1186/s12872-024-03893-0.

## Introduction

Myocardial infarction (MI), a leading cause of death worldwide, is characterized by cardiac blood flow interruption and results irreversible death of cardiomyocytes (CMs) [1, 2]. CMs is difficult to proliferate after myocardial tissue injury, and its loss underlies the fundamental cause of heart failure post MI. Although available medical and device-based therapies could improve cardiac function, incidences of heart failure still remain quite high [3]. Therefore, exploring an effective way for myocardial regeneration following cardiac injury has become a hot topic in current research.

Macrophages, as the core regulatory cell population of inflammatory responses, play an important regulatory role in the process of cardiac repair and remodeling post-MI [4–6]. Macrophages are divided into two typical subtypes: classically activated macrophages (M1) and alternatingly activated macrophages (M2) [7, 8]. After MI, M1 macrophages are responsible for removing cell fragments and dead cells clearance and keep predominant during the first inflammatory stage (within 5 days), whereas M2 macrophages dominate later (the injury-resolution phase) to promote collagen deposition, scar maturation and angiogenesis [9, 10]. The excessive activity of M1 macrophages have been shown to promote cardiomyocytes death [11], inhibit myocardial regeneration [12], and inhibition of M1 macrophages could alleviate adverse ventricular remodeling [13]. However, the underlying mechanisms of M1 macrophages on myocardial regeneration require further exploration.

Exosomes are small-membrane fragments with a diameter of 30–150 nm, actively secreted by most cell types and act as mediators of intercellular communication by transferring intracellular cargoes, including enzymes, DNA, and non-coding RNAs. The role of exosomes or extracellular vesicles derived from M1 macrophages has been recently elucidated. For instance, M1 macrophages derived from adipose tissue could secrete exosome,richly of miR-155,  leading to insulin resistance in mice [14]. Our previous study had revealed that M1 macrophage-derived exosomes (M1-Exos) exerted an anti-angiogenic effect, which accelerated MI injury and inhibited cardiac repair by delivering proinflammatory miR-155 [15]. Nevertheless, the effect of M1-Exos on cardiomyocytes regeneration during MI remains unclear.

In the present study, we investigated the effect of M1-Exos on cardiomyocytes regeneration. Our findings revealed that M1-Exos transferred specific miR-155 into cardiomyocytes subsequently depressing the IL-6R/JAK/STAT3 signaling pathway and affecting myocardial regeneration and cardiac repair after MI.

## Materials and methods

### Experimental animals

Male C57BL/6J mice aged 8–10 weeks were purchased from Guangzhou Dean Gene Technology Co., Ltd (Guangzhou, China). All experimental protocols complied with the Guide for the Care and Use of Laboratory Animals published by the US National Institutes of Health (NIH) and the Animal Care and Use Committees of Universities and hospitals. C57BL/6 mice were housed in a pathogen-free animal facility and allowed to eat and drink ad libitum. After 1 week of accommodation to environmental conditions, they were anesthetized with 2.0% isoflurane during MI model operation. After the study, all the mice were first anesthetized by allowing them to inhale 2.0% isoflurane and subjected to cervical dislocation.

### MI model

The MI model was established by ligating the left anterior descending (LAD) coronary artery as previously described. Briefly, the mice were anesthetized with 2.0% isoflurane, and ventilated through an endotracheal tube connected to a rodent ventilator, followed by permanent ligation of the left anterior descending (LAD). The successful establishment of the MI model was confirmed by observation of myocardial whitening around the ligation area and ST-segment elevation in the electrocardiogram. Mice in the Sham group underwent the same surgical operation, except for the LAD ligation. Immediately after LAD ligation, small Murine lentivirus LV-NC and LV-miR-155 was injected into the peri-infarct area with insulin injection needle. LV- NC and LV-miR-155 sequences as following:

**Table:** 

Sequence	
LV-NC	TTCTCCGAACGTGTCACGT
LV-mmu-miR-155	TTAATGCTAATTGTGATAGGGGT

### Induction of M1 macrophages

RAW 264.7 mouse macrophages (SCSP-5036) were cultured in DMEM (Gibco, C11995500BT) supplemented with 10% FBS (Biological Industries, 1,913,444) and 1% penicillin and streptomycin (Gibco, 15,140,122). When M0 macrophages reached 70–80% confluency, culture medium was replaced with that containing 5% exosome-depleted FBS (ViVaCell, C38010050). M0 macrophages were stimulated with mouse 50 ng/mL GM-CSF (Sino Biological, 51,048-MNAH) and 20 ng/mL IFN-γ (Sino Biological, 50,709-MNAH) for 24 h to induce M1 macrophages. After the treatment, macrophages and culture supernatants were collected for analysis.

### Isolation of exosomes from macrophages

The medium of M0 macrophages and M1 macrophages was collected separately, and the exosomes in the medium were isolated by differential ultracentrifugation. The collected medium was centrifuged in 50 mL centrifuge tubes at 4 °C for 300×g for 15 min, 2,000×g for 30 min, and 10,000×g for 60 min. The supernatant was transferred and centrifuged at 100,000×g for 90 min at 4 °C. The final obtained particles were suspended in PBS. The morphology of exosomes was observed under a transmission electron microscope (TEM, Hitachi). The size of exosmes was analyzed by nanoparticle tracking analysis (NTA, NanoSight NS300 Malvern). The surface markers CD63, CD9 and Calnexin were analyzed by Western blot analysis.

### Culture and treatment of primary cardiomyocytes

Isolation of Neonatal cardiomyocytes were isolated from 1-to-3-day-old neonatal C57BL/6 mice [16, 17]. Briefly, the animals were euthanized by decapitation, and the hearts were excised and washed in an ice-cold PBS. The ventricular tissue was pressed into thin tissue pieces, which were digested with 0.125% trypsin (Gibco, 25,200,072) at 4 °C for 12–13 h. Then the tissue was digested with 15 mg/mL type II collagenase (Gibco, 17,101,015) at 120 rpm, 37 °C for 30 min. Then, the cell suspension was seeded into twice in 100-mm culture dishes and cultured twice for 1.5 h (37 °C, 5% CO_2_, 95% air) to isolate cardiomyocytes and cardiac fibroblasts. The isolated cardiomyocytes were seeded in six-well plate and incubated in DMEM containing 10% FBS, 1% penicillin-streptomycin, and 100 μm Brdu for 48 h (37 °C, 5%CO2, 95% air). The cardiomyocytes were treated with 50 ng/mL IL-6.

### Uptake and treatment of exosomes

Cellular uptake of exosomes in cardiomyocytes was explored by labeling with PKH67 (Red, Sigma) using a commercial kit according to the manufacturer’s instructions. Exosomes were resuspended in 200 µL of diluent C and then 300 µL of Diluent C and 4 µL PKH67 dye were added. The mixture was incubated at room temperature for 5 min. Subsequently, 2mL 1% bovine serum albumin (BSA, Sigma-Aldrich) was added to bind excess dye. An appropriate amount of medium was added, and the mixture was centrifuged twice at 100,000×*g* for 90 min at 4 °C to remove unbound dye. The labeled exosomes were resuspended in DMEM containing 5% exosome-depleted FBS, added to cardiomyocytes at 2 × 10^9^/mL, and placed in a cell incubator (37 °C, 5% CO_2_, 95% air) in the dark for 10 h. After the cells were fixed with 4% paraformaldehyde, the cytoskeleton was stained with phalloidin-iFluor488 (Abcam, ab176753) at room temperature for 30 min in the dark, and the nuclei were stained with 4′,6-diamidino-2-phenylindole (DAPI) at room temperature for 10 min in the dark. After the excess antibody was washed, an anti-fluorescence quencher was added, and the uptake was observed and photographed under a laser confocal microscope.

For the treatment, exosomes (2 × 10^9^/mL) were added to DMEM containing 5% exosome-depleted FBS and co-cultured with cardiomyocytes for 24 h.

### Cardiomyocyte transfection

The mmu-miR-155 mimic and a negative control were purchased from Suzhou GenePharma Co.,Ltd. Cardiomyocytes were transfected with 100 nM miR-155 mimic and the negative control (NC) according to the manufacturer instructions. 48 h after transfection, the cells were harvested for subsequent experiments. miR-155 mimic and NC sequences as following:

**Table:** 

negative control	sense	UUCUCCGAACGUGUCACGUTT
	antisense	ACGUGACACGUUCGGAGAATT
mmu-miR-155 mimic	sense	UUAAUGCUAAUUGUGAUAGGGGU
	antisense	CCCUAUCACAAUUAGCAUUAAUU

### Immunocytochemistry

Cardiomyocytes were seeded into 24-well plates, washed with PBS, and fixed in 4% paraformaldehyde (Beyotime). Next, the cells were permeated with 0.5% Triton X-100 in PBS, blocked with 5% BSA and incubated with primary antibodies pH3 (CST, 9713, 1:500, Rabbit), Ki67 (CST, 9129, 1:500, Rabbit), and α-actinin (CST, 69,758, 1:500, Mouse) over-night. After that, the cells underwent 1 h incubation with Alexa Fluor® 488-conjugated goat anti-rat antibody (Abcam, ab150165,1:500) or Alexa Fluor® 647-conjugated goat anti-rabbit antibody (Abcam, ab150079, 1:500). DAPI was added and incubated at room temperature for 10–15 min. Finally, images were observed and taken under a fluorescent inverted microscope.

### Immunohistochemistry

Heart tissue was processed for paraffin embedding, sectioned, and deparaffinized. Sections were blocked in 3% BSA and labelled with unconjugated primary antibodies against PH3(CST, 9713, 1:500, Rabbit) and α-actinin (CST, 69758,1:200, Mouse). The nuclei were counterstained with DAPI and observed under a microscope.

### RNA extraction and quantification

Total mRNA extraction was performed according to standard protocols. A PrimeScript RT Reagent Kit (Takara) was used to perform reverse transcription for cDNA production. Quantitative reverse transcription polymerase chain reaction (qRT-PCR) was conducted using TB Green Premix Ex Taq II (Tli RNaseH Plus) kit. The 2 − ∆∆CT (∆∆ CT = ∆ CT (experimental) − ∆ CT (negative control)) method was employed to determine the level of IL-6R. The primers of IL-6R used were as follows: F:5’-CCTGAGACTCAAGCAGAAATGG-3’, R:5’-AGAAGGAAGGTCGGCTTCAGT-3’.

### Dual luciferase reporter assays

The potential molecular target of miR-155 was predicted through http://www.targetscan.org (TargetScan 4.2). As a potential target, we focused on IL-6R, which is part of the JAK/STAT pathway that plays a significant role in cardiomyocyte proliferation. HEK293T cells were cotransfected with either control or miR-155 plasmids, using Lipofectamine 2000 following the manufacturer’s protocols. A double-luciferase reporter activity (GeneCopoeia) system was used to compare the activity of Renilla and dual-luciferase reporters 48 h after transfection.

### Immunoblotting analysis

Total protein was extracted from NRCMs and exosomes using RIPA lysis buffer and protease inhibitor cocktail (Beyotime). The protein concentration was measured using a BCA protein kit (Beyotime). Proteins were separated by 10% sodium dodecyl sulfate polyacrylamide gel electrophoresis (SDS-PAGE) and transferred onto polyvinylidene difluoride (PVDF) membranes (Bio-Rad, China). Next, the membranes were incubated overnight at 4℃ with primary antibodies CD63 (Abcam, ab193349, 1:1000, Mouse), CD9 (Abcam, ab92726, 1:1000, Rabbit), anti-CD81 (Santa Cruz, sc-166,029, 1:1000, Mouse), Calnexin (Proteintech, 10427-2-AP, 1:1000, Rabbit), p-JAK2 (CST, 3771, 1:1000, Rabbit), p-STAT3 (CST, 9145, 1:1000, Rabbit), IL-6R (Santa Cruz, sc-373,708, 1:500, Mouse) and GAPDH (CST, 5174, 1:1000, Rabbit). Secondary antibodies were prepared using 5% skim milk (1:5000).

### Statistical analysis

Statistical analysis of data was performed on GraphPad Prism 8.0 software, and all data were expressed as mean ± standard error. Comparisons between two groups were performed using T test, and comparisons between multiple groups were performed using one-way ANOVA or two-way ANOVA. If statistical significance was found, the Bonferroni method was used for further statistical analysis, and the difference was considered to be statistically significant when *P* < 0.05.

## Results

### Induction and identification of M1 macrophage-derived exosomes (M1-Exos)

**Fig. 1:**
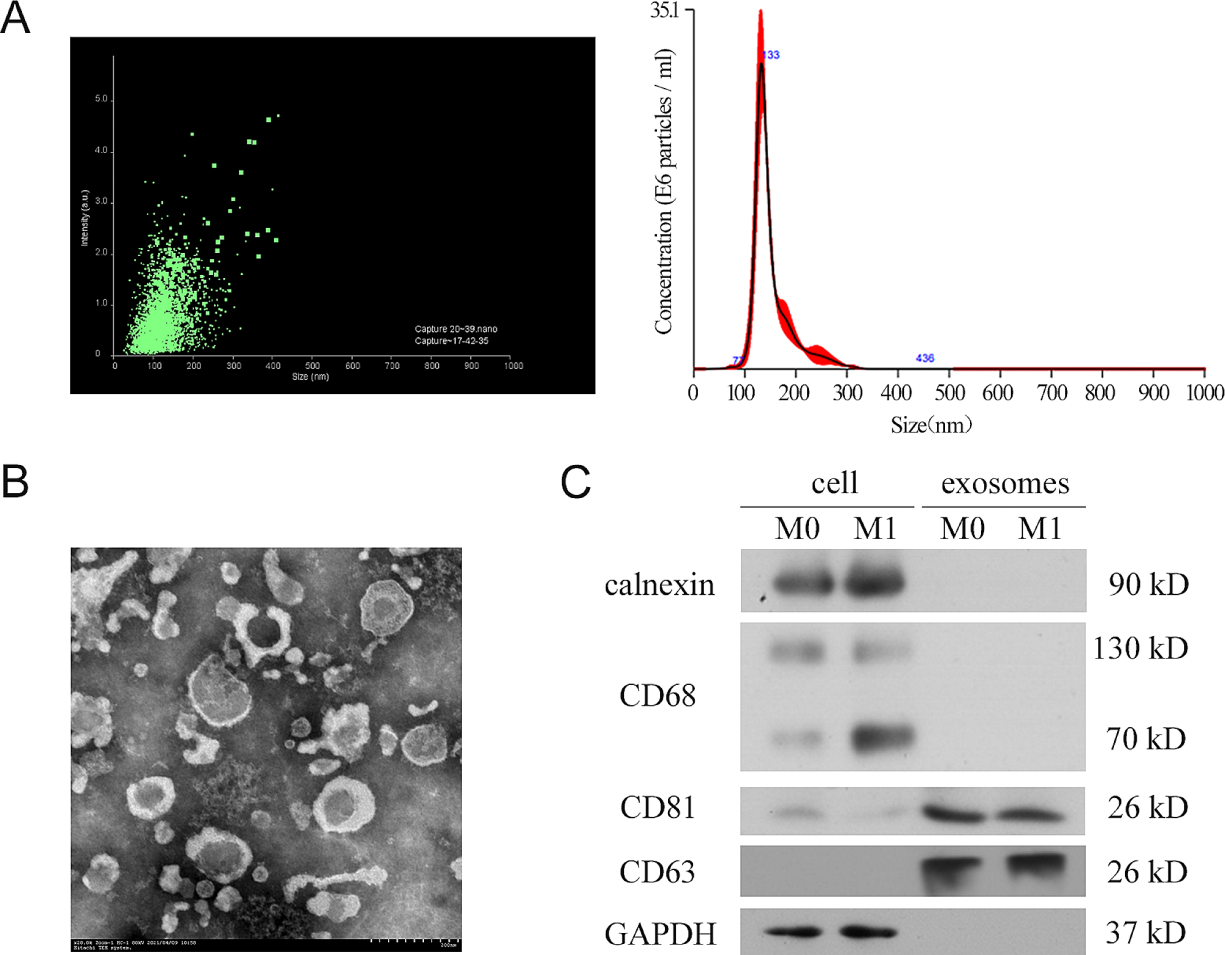
Induction and identification of M1-Exos **A**) The particle size and concentration of isolated exosomes were detected using nanoparticle tracking analysis. **B**) The size and shape of macrophage-derived exosomes were observed by transmission electron microscopy. Scale bar: 200 nm. **C**) Immunoblotting was performed with macrophage marker proteins calnexin and CD68, and exosome marker proteins CD81 and CD63. GAPDH served as a loading control. Each experiment in this figure was replicated more than three times

The M0- and M1- macrophage exosomes were isolated in accordance with differential ultracentrifugation. The particle size and concentration of the isolated exosomes were measured by nanoparticle tracking analysis (NTA), showing that almost all particles were between 77 and 200 nm in size, with a peak at 133 nm (Fig. 1A). Meanwhile, the morphology of the isolated exosomes was observed by transmission electron microscopy (TEM), indicating the size of exosome particles was approximately 100–150 nm, and they were saucer-like vesicles (Fig. 1B). The CD81 and CD63, as biomarker proteins of exosomes, were only identified in exosomes derived from M0 and M1 macrophage by immunoblotting analysis (Fig. 1C). Calnexin, a protein derived from the endoplasmic reticulum, and the macrophage marker protein CD68 were only expressed in M0 and M1 macrophages but not in exosomes (Fig. 1C). The morphology, particle size, and protein markers of the isolated particles were consistent with the characteristics of exosomes.

### M1-Exos inhibited cardiomyocyte proliferation

**Fig. 2:**
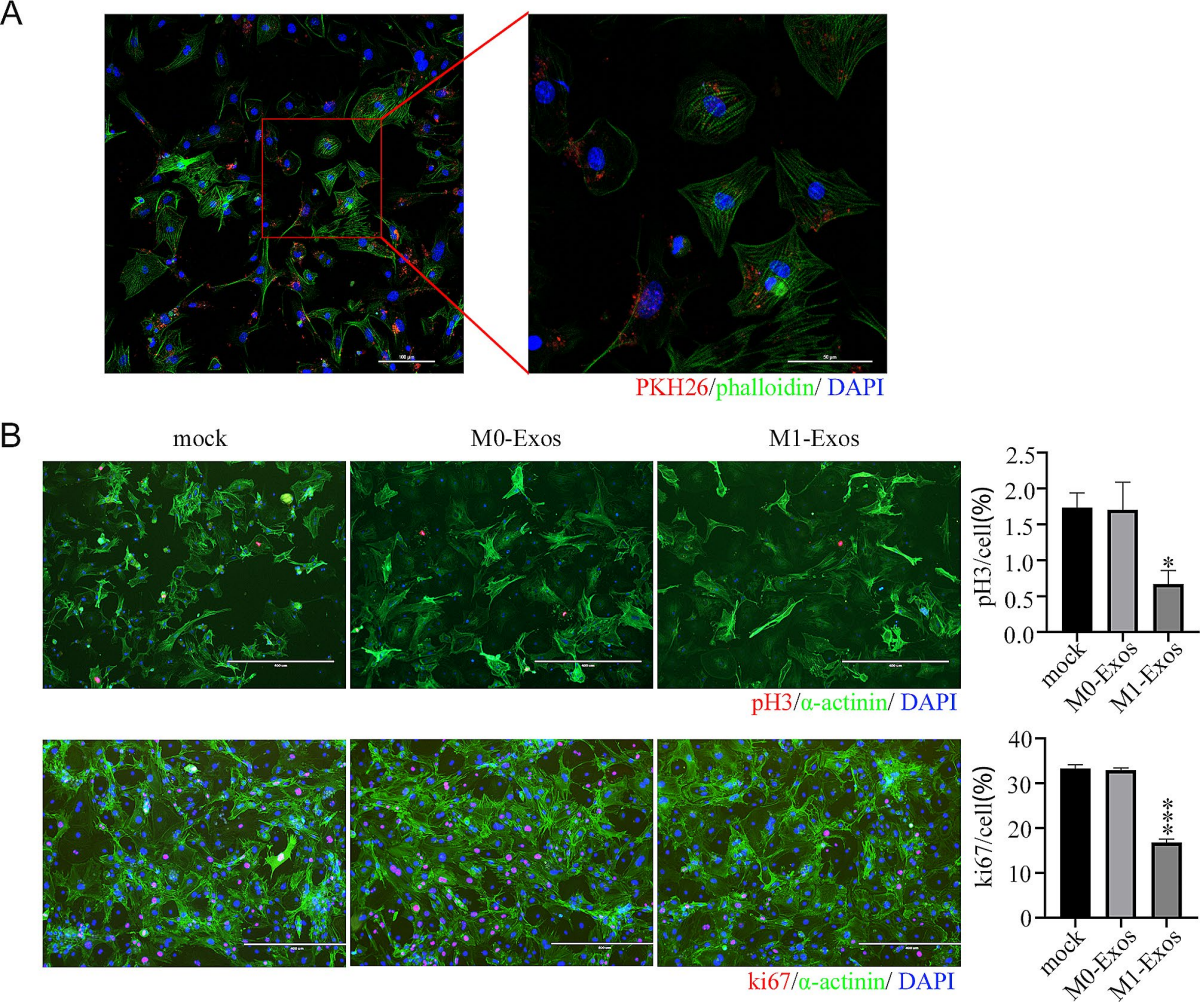
Inhibition of cardiomyocyte proliferation by M1-Exos **A**) After PKH26-labeled exosomes (red) were added to cardiomyocytes at a concentration of 2 × 10^9^/mL for 10 h, the uptake of exosome was observed by confocal laser microscopy. Scale bar: 100 μm. **B**) M0-Exos and M1-Exos were added to cardiomyocytes at a concentration of 2 × 10^9^/mL for 24 h, and the number of cardiomyocytes labeled with pH3 or ki67 (red) was observed by immunocytochemistry. Scale bar: 400 μm. Each experiment in this figure was replicated more than three times. *P < 0.05, ***P < 0.001, M1-Exos group compared with M0-Exos group

Primary cardiomyocytes were treated with the labeled M1-Exos which uptake was detected by confocal laser microscope. PKH26-labeled macrophage-derived exosomes (red) were observed in cardiomyocytes and accumulated around the nucleus (Fig. 2A), suggesting primary cardiomyocytes could take up macrophage-derived exosomes. After co-cultured for 24 h with the labeled M1-Exos, the number of positive cells for both phospho-pH3 and ki67 markers in cardiomyocytes was significantly reduced compared with the mock and the M0-Exos groups (Fig. 2B). This finding indicated that M1-Exos inhibited the proliferation ofcardiomyocytes.

### M1-Exos delivered miR-155 to cardiomyocytes

**Fig. 3:**
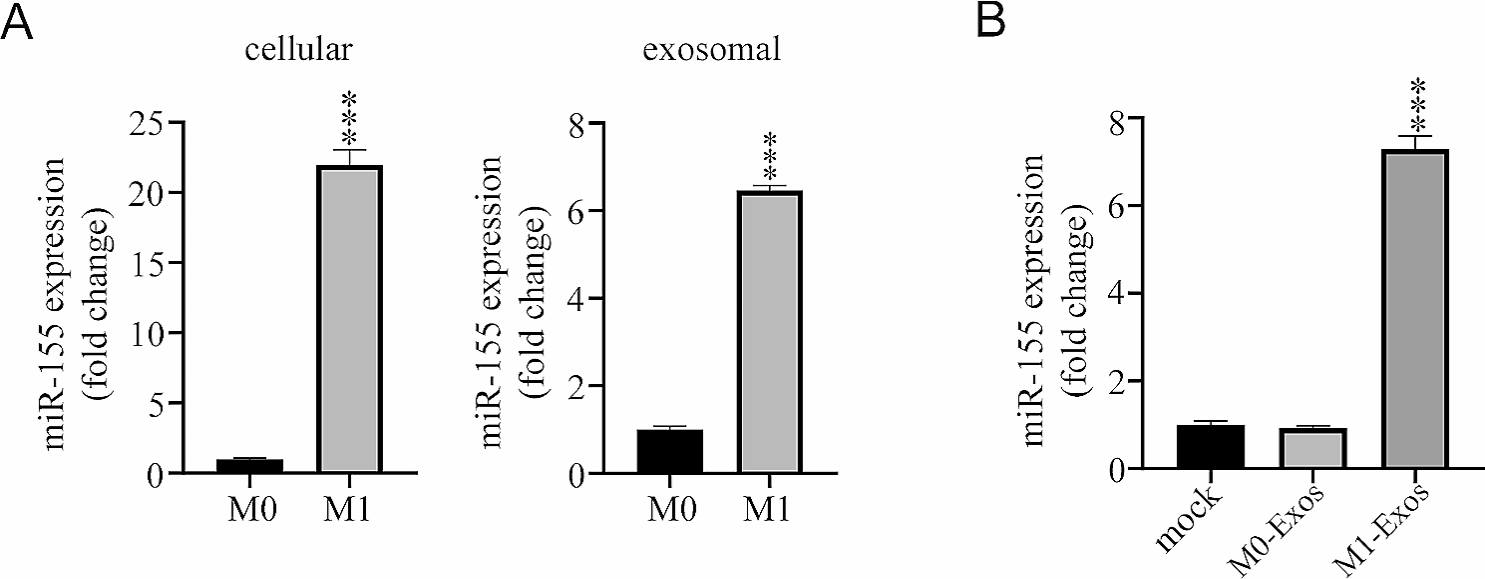
Delivery of miR-155 to Cardiomyocytes through M1-Exos **A**) M1 macrophages were treated with 50 ng/mL GM-CSF and 20 ng/mL IFN-γ for 24 h. The expression of miR-155 in cells (left) and exosome (right) was detected by qRT-PCR. **B**) After M0-Exos and M1-Exos were added to cardiomyocytes at a concentration of 2 × 10^9^/mL for 24 h, the expression of miR-155 in cardiomyocytes was detected by qRT-PCR. Each experiment in this figure was replicated more than three times. ***P < 0.001, M1 vs. M0, or M1-Exos vs. M0-Exos treatment

As a particular marker and mediator miRNA for M1 macrophages differentiation, miR-155 is richly expressed in M1-Exos. The present study yielded similar results. The expression of miR-155 in M1 macrophages and M1-Exos was higher than that in M0 macrophages and M0-Exos (Fig. 3A). The expression of miR-155 in cardiomyocytes treated with M1-Exos was significantly increased compared with that in the mock and the M0-Exos groups (Fig. 3B), suggesting M1-Exos were rich in miR-155 and carried miR-155 to primary cardiomyocytes.

### miR-155 inhibited cardiomyocyte proliferation

**Fig. 4:**
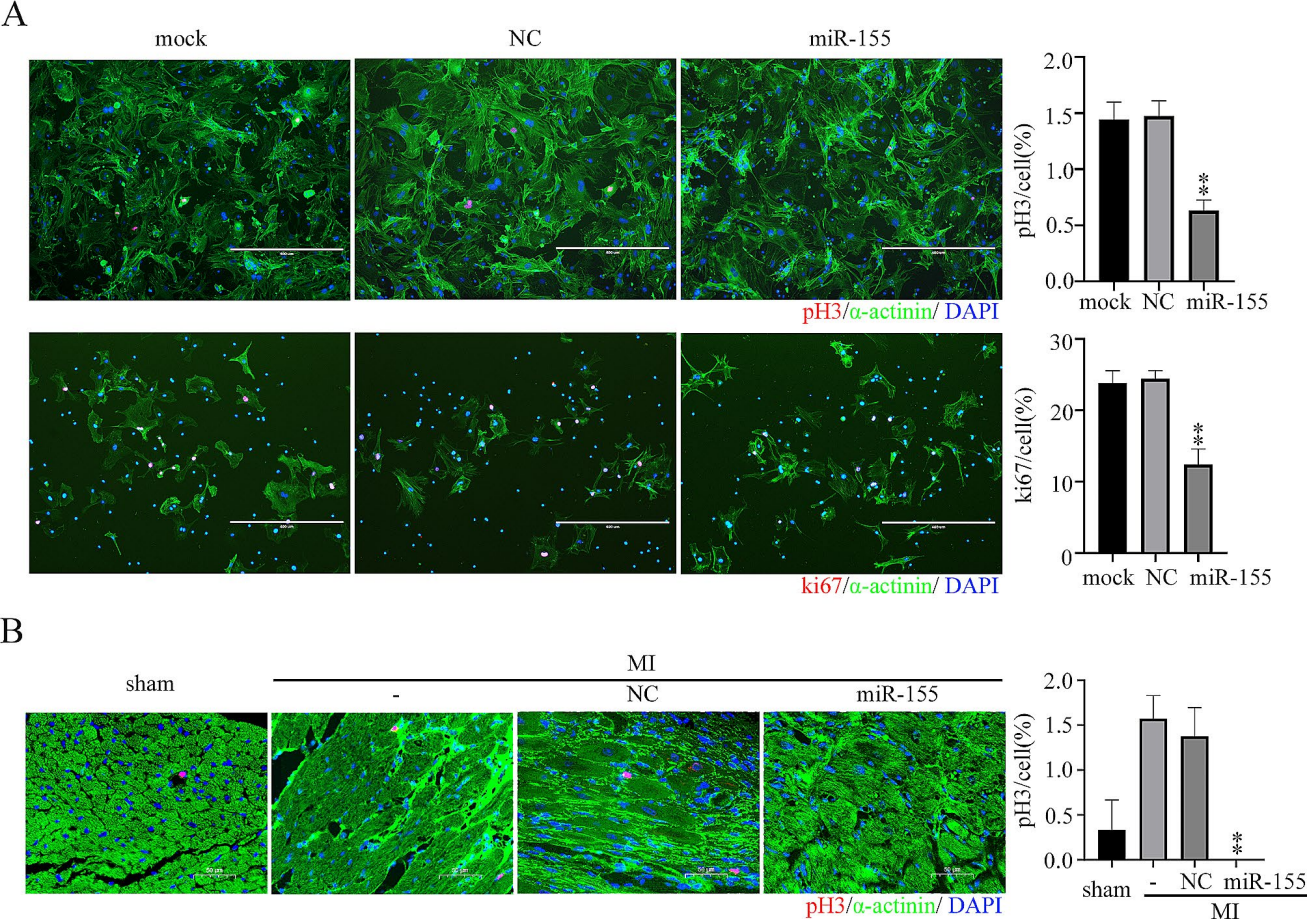
Inhibition of cardiomyocyte proliferation by miR-155 **A**)Negative control or miR-155 mimic was transfected into cardiomyocytes at 100 nM for 48 h, and the number of cardiomyocytes labeled with pH3 or ki67 (red) was observed by immunocytochemistry. Scale bar: 400 μm. **B**) After the mouse MI model was constructed, 50 µl of LV-mmu-miR-155 was injected into the peri-infarct tissue. The hearts were removed for immunofluorescence after 14 days, and the number of pH3-labeled Cardiomyocytes was observed. Each experiment in this figure was replicated more than three times. **P < 0.01, miR-155 vs. NC treatment

To test the effect of miR-155 on cardiomyocytes proliferation, miR-155 mimic was transfected into primary cardiomyocytes. The positive number of cardiomyocytes marked by pH3 or ki67 in the miR-155 group was significantly reduced compared with that in the mock and the negative control groups (Fig. 4A).

In vivo, LV-miR-155 was injected into the peri-infarct tissue of MI mice. The positive number of cardiomyocytes marked by pH3 in the miR-155 group was significantly decreased compared with that in the MI and negative control groups (Fig. 4B).The data demonstrated that miR-155 inhibited cardiomyocyte proliferation and myocardial regeneration in vitro and in vivo.

### miR-155 targeted IL-6R mRNA

**Fig. 5:**
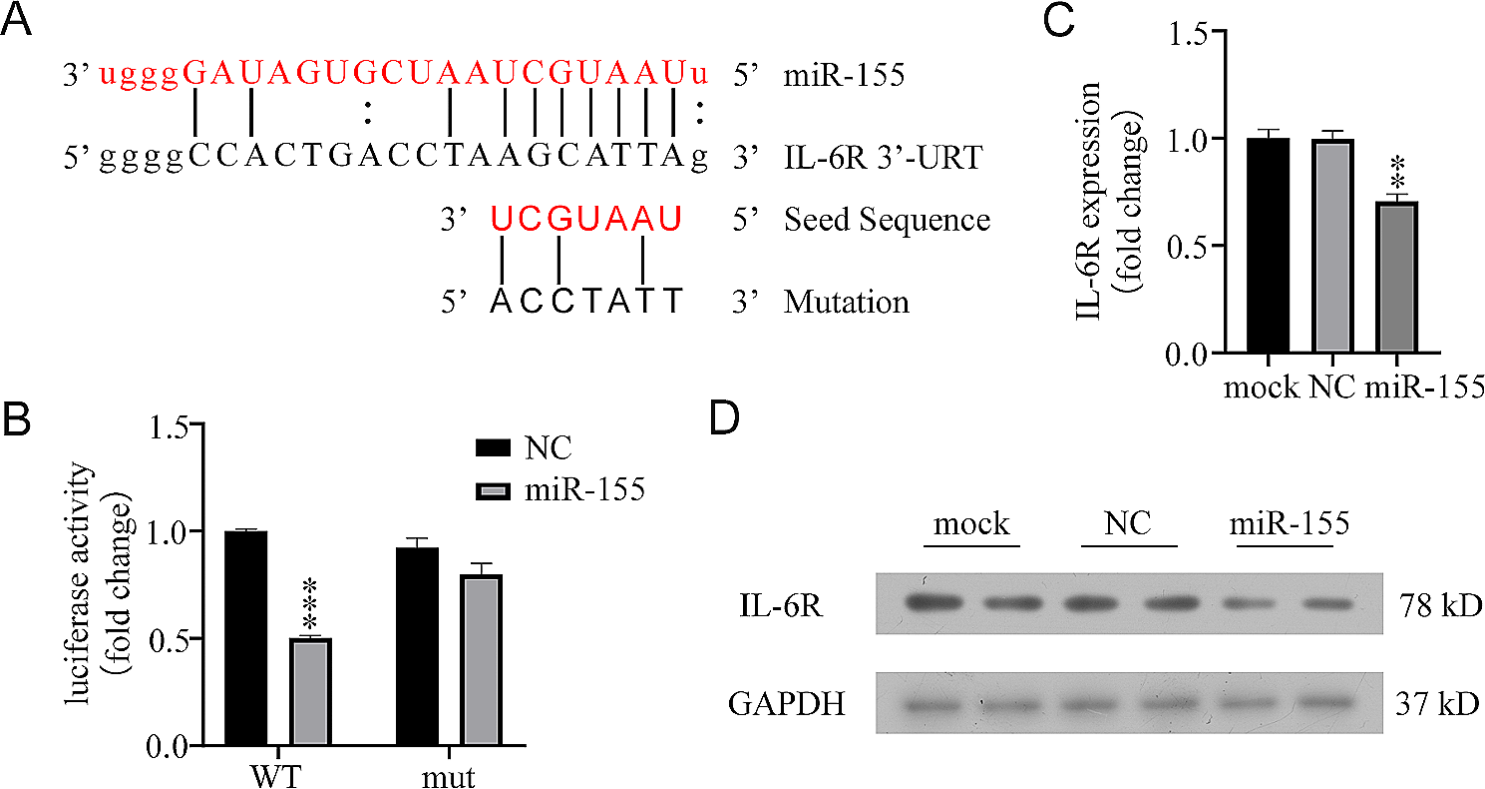
IL-6R as a novel target gene of miR-155 **A**) Bioinformatics analysis suggested that IL-6R is a new target gene of miR-155. **B**) 20 nM of negative control or miR-155 mimic and 150 ng of pmirGLO plasmid containing WT or 3′ -UTR mutation of target gene were co-transfected into HEK293T cells, and dual luciferase activity was detected 48 h later. ***P < 0.001, miR-155 mutant vs. miR-155 WT. **C**) Negative control or miR-155 mimic was transfected into Cardiomyocytes at 100 nM, and the mRNA expression of IL-6R in cardiomyocytes was detected by qRT-PCR after 48 h. **P < 0.01, miR-155 vs. NC treatment. **D**) Negative control or miR-155 mimic was transfected into cardiomyocytes at 100 nM, and the IL-6R in cardiomyocytes was analyzed by immunoblotting after 48 h. Each experiment in this figure was replicated more than three times

Next, the specific molecular mechanism by which miR-155 inhibited cardiomyocyte proliferation was elucidated through bioinformatics analysis. IL-6R was predicted as a potential target gene of miR-155 (Fig. 5A). The dual luciferase reporter assays confirmed that miR-155 could significantly inhibit the WT 3′ -UTR of IL-6R but not the mutant 3′ -UTR (Fig. 5B). Further, the mRNA level (Fig. 5C) and protein expression (Fig. 5D) of IL-6R in the cardiomyocytes transfected with miR-155 were decreased compared with those in the mock and negative control groups. These results indicated that IL-6R was a direct target gene of miR-155.

### miR-155 antagonized IL-6-induced cardiomyocyte proliferation by inhibiting IL-6R/JAK/STAT pathway

**Fig. 6:**
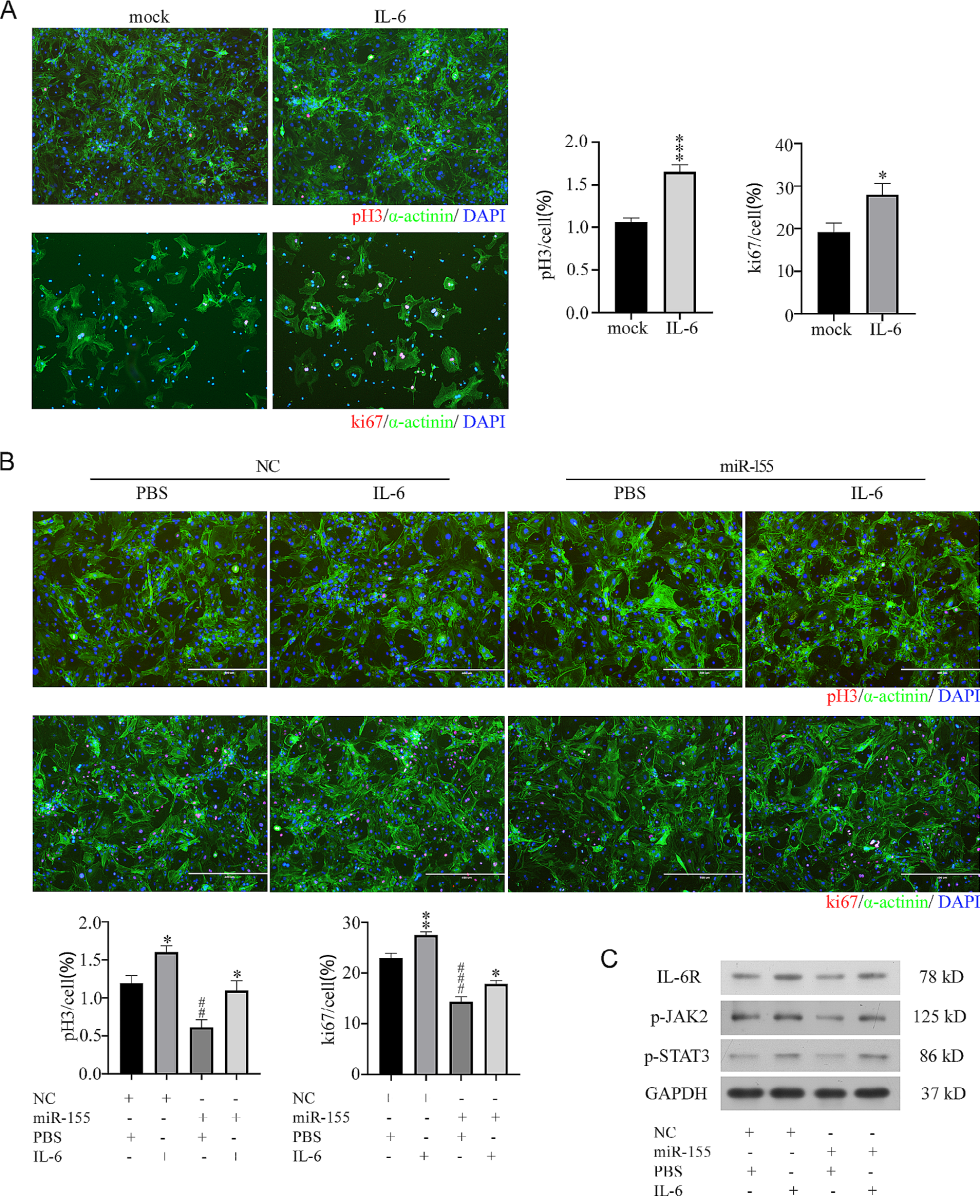
Inhibition of IL-6R/JAK/STAT pathway by miR-155 **A**) IL-6 was added to cardiomyocytes at 50 ng/mL for 24 h, and the number of cardiomyocytes labeled with pH3 or ki67 was observed by immunocytochemistry. Scale bar: 400 μm. *P < 0.05 and ***P < 0.001, IL-6 vs. mock treatment. **B**) Negative control or miR-155 mimic transfected cardiomyocytes at 100nM for 24 h, and then IL-6 was added at 50 ng/mL for 24 h. The number of cardiomyocytes labeled with pH3 or ki67 was observed by immunocytochemistry. Scale bar: 400 μm. *P < 0.05 and **P < 0.01, IL-6 vs. PBS treatment. ##*P* < 0.01 and ###*P* < 0.001, miR-155 vs. NC treatment. **C**) Negative control or miR-155 mimic transfected cardiomyocytes at 100 nM for 24 h, and then IL-6 was added for 24 h. Immunoblotting was performed with IL-6R, phospho-Jak2, and phospho-Stat3 antibodies. GAPDH was used as a loading control. Each experiment in this figure was replicated more than three times

The IL-6/JAK/STAT pathway plays a key role in cardiomyocyte proliferation and cardiac repair. Here, whether IL-6-induced cardiomyocyte proliferation was regulated by miR-155 was explored. The positive number of cardiomyocytes marked by pH3 or ki67 in the IL-6 treatment group was significantly increased compared with that in the control group, which verified the pro-proliferation effect of IL-6 on cardiomyocytes (Fig. 6A). miR-155 significantly decreased the positive number of IL-6-induced cardiomyocytes marked by pH3 or ki67 (Fig. 6B). In addition, IL-6 treatment markedly increased the protein levels of IL-6R, phospho-JAK2, and phospho-STAT3, while miR-155 antagonized this effect of IL-6 (Fig. 6C). Therefore, miR-155 antagonized IL-6-induced cardiomyocyte proliferation by inhibiting the IL-6R/JAK/ STAT pathway.

## Discussion

Acute MI leads to the loss of about 25% of cardiomyocytes from the left ventricle, corresponding to up to 1 billion cells [18]. Due to cardiomyocytes hardly regenerating, the lack of cardiomyocytes is the main reason for poor ventricular remodeling and heart failure post MI. In this study, we demonstrated that activated macrophages secreted miR-155-containing exosomes which were taken up by cardiomyocytes. The miR-155-enriched exosomes suppressed cardiomyocyte proliferation and myocardial regeneration following MI by targeting IL-6R and inhibiting the JAK/STAT3 signaling pathway. Our findings provided the groundwork for research on macrophage exosomes, aimed towards a greater understanding of their role in regulating cardiomyocyte proliferation.

In the early inflammatory stage after myocardial infarction, a large number of inflammatory cells accumulate in the infarcted site, and monocytes differentiate into macrophages, which could be polarized into M1 macrophages and M2 macrophages. M1 macrophages are believed to play an inhibitory role in cardiac repair after myocardial infarction, while M2 macrophages are often considered to have anti-inflammatory functions, such as promoting angiogenesis, cell proliferation, and tissue repair [19]. Regulating the balance switch between pro-inflammatory M1 macrophages and anti-inflammatory M2 macrophages can affect the inflammatory response at the site of myocardial infarction, thereby regulating the process of cardiac repair [20]. The previous study had showed that the appropriate conversion of M1 macrophages to M2 macrophages could promote the function of collagen deposition and endothelial cell tubule formation, and accelerate wound healing [21]. It has been reported that M1 macrophages could aggravate left ventricular remodeling after MI by secreting exosomes [22]. Our recently study indicated that M1-Exos inhibited endothelial cell regeneration, accelerated MI injury, and prevented cardiac repair. However, the effect of M1-Exos on cardiomyocytes proliferation is unclear. Therefore, in the present study, we first explore the effect of M1-Exos on cardiomyocyte proliferation and its underlying mechanism. Our results revealed that M1-Exos treatment suppressed cardiomyocyte proliferation and myocardial regeneration in vitro and vivo.

MiR-155, a known proinflammatory regulator of myeloid and lymphoid immune cell function, was induced during activation of macrophages [23]. MiR-155 is an important regulator of immune inflammation and involved in the regulation of pathophysiological processes, such as cardiovascular disease and immune inflammation [24]. The expression of miR-155 was upregulated in exosomes of activated macrophages and delivered to fibroblasts, which inhibited the proliferation of fibroblasts and aggravated cardiac function post MI [22]. MiR-155 also inhibited endothelial cell proliferation, tubule formation, cell migration, and other functions, thereby further inhibiting cardiac repair [15]. This study indicated that M1-Exos contained richly miR-155, which could be delivered to cardiomyocytes, thereby inhibiting cardiomyocyte proliferation. miR-155 plays the inhibitory roles in regulating myocardial regeneration and cardiac repair after myocardial infarction. Inhibition of miR-155 could promote the polarization of macrophages to M2 macrophages [25]. It was observed that inhibition of miR-155 reduced myocardial damage during viral myocarditis, and long-term inhibition of miR-155 even improved survival and cardiac function [23]. Therefore, miR-155 may be an important target for the treatment of myocardial infarction, and inhibition of miR-155 may be a potential strategy for promoting myocardial regeneration and cardiac repair.

IL-6 is necessary for myocardial regeneration and cardiac repair [26]. The pro-proliferation effect of IL-6 on neonatal mouse cardiomyocytes is mediated by STAT3, the major downstream effector of IL-6 [27]. The JAK/STAT3 signaling pathway in the heart has multiple cellular functions, including myocardial differentiation, cell cycle re-entry after injury and anti-apoptosis under pathological conditions [28]. Thus, modulating JAK/STAT3 signaling pathway has great potential to regulate myocardial regeneration. The cardiomyocyte regeneration in zebrafish was confirmed to be promoted by the JAK/STAT3 signaling pathway, and inhibition of Stat3 expression could inhibit myocardial regeneration [29]. In the present study, IL-6R was predicted and confirmed to be a novel target gene of miR-155. miR-155 could inhibit cardiomyocyte proliferation by antagonizing the IL-6R/JAK/STAT3 pathway.

**Fig. 7:**
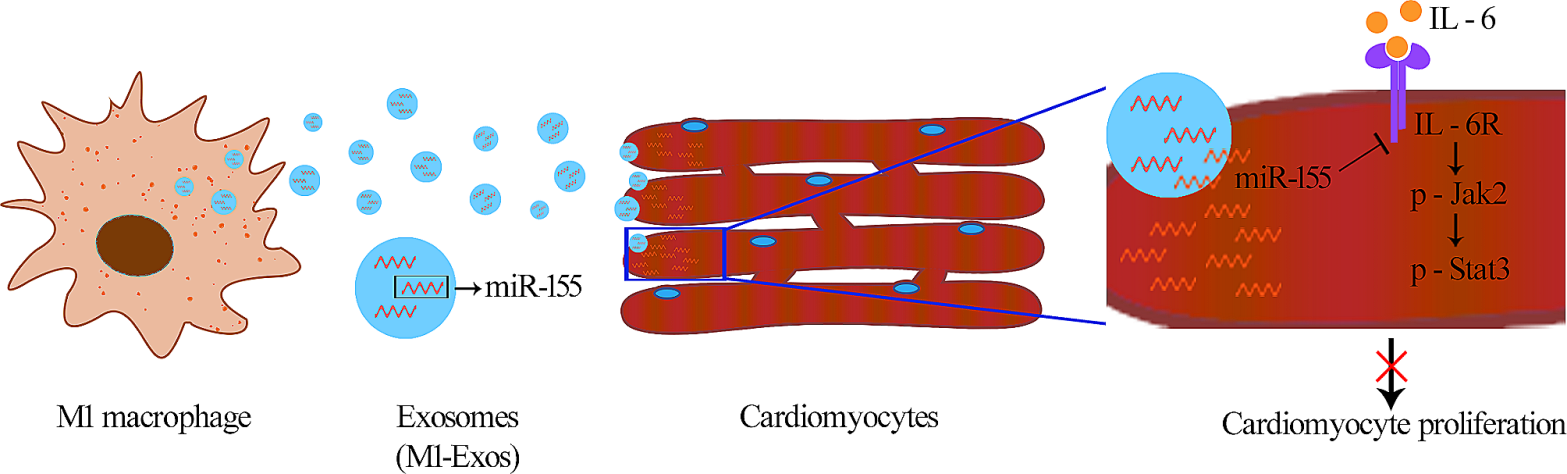
Working mode diagram When macrophages differentiated into M1 macrophages which secrete M1-Exos in large amount. M1-Exos carried miR-155 into cardiomyocytes and targeted IL-6R, thus inhibiting the IL-6R/JAK/STAT pathway, which resulted in the inhibition of cardiomyocyte proliferation

In conclusion, our current study provided evidences indicating that M1-Exo inhibited myocardial regeneration via delivering miR-155 to cardiomyocytes and restraining IL-6R/JAK/STAT3 pathway activation following MI (Fig. 7), which provided the groundwork for research on macrophage exosomes aimed toward greater a understanding of their role in regulating cardiomyocyte proliferation. More studies will be performed to investigate whether eliminating M1-Exos could be manipulated to improve cardiac injury post MI and further evaluate the therapeutic potential of M1-Exos-mediated processes to aggravating cardiac repair as revealed in this study.

### Electronic supplementary material

Below is the link to the electronic supplementary material.

Supplementary Material 1

Supplementary Material 2

## Data Availability

Data is provided within the manuscript or supplementary information files. Also, the data that support the findings of this study are available from the corresponding author upon reasonable request.
