# Supplementary material for: M1 macrophage-derived exosomes inhibit cardiomyocyte proliferation through delivering miR-155
Source: BMC Cardiovasc Disord. 2024 Jul 16;24:365. doi: 10.1186/s12872-024-03893-0 (PMC11251235; doi:10.1186/s12872-024-03893-0)
Supplement: Supplementary file 1 — Supplementary Material 1 [file 12872_2024_3893_MOESM1_ESM.docx]

**Figure 1**

**Figure 5**

**Figure 6**
