# Supplementary material for: M1 macrophage-derived exosomes inhibit cardiomyocyte proliferation through delivering miR-155
Source: BMC Cardiovasc Disord. 2024 Jul 16;24:365. doi: 10.1186/s12872-024-03893-0 (PMC11251235; doi:10.1186/s12872-024-03893-0)

**Fig1.Calnexin**


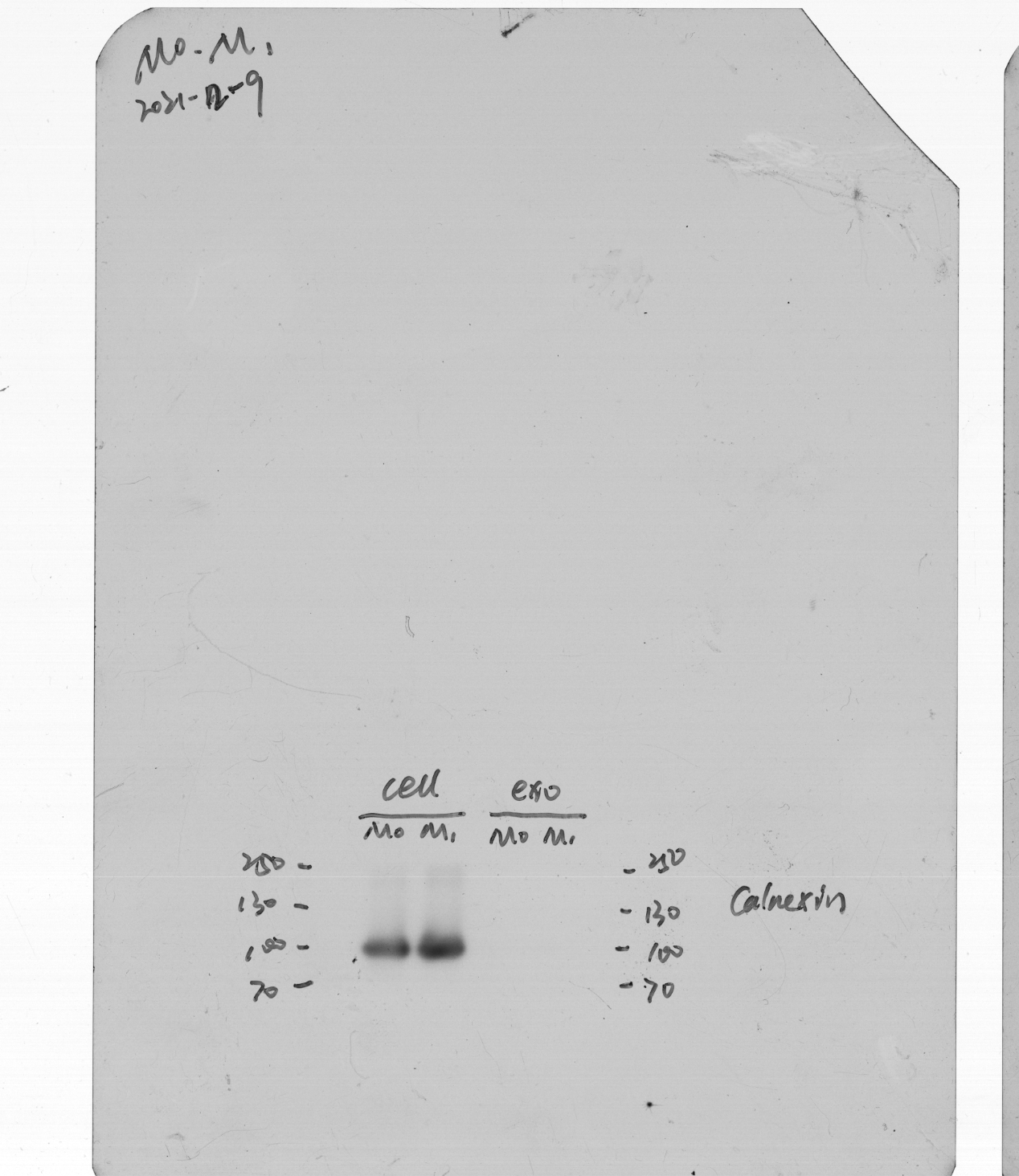


**Fig1C.CD68
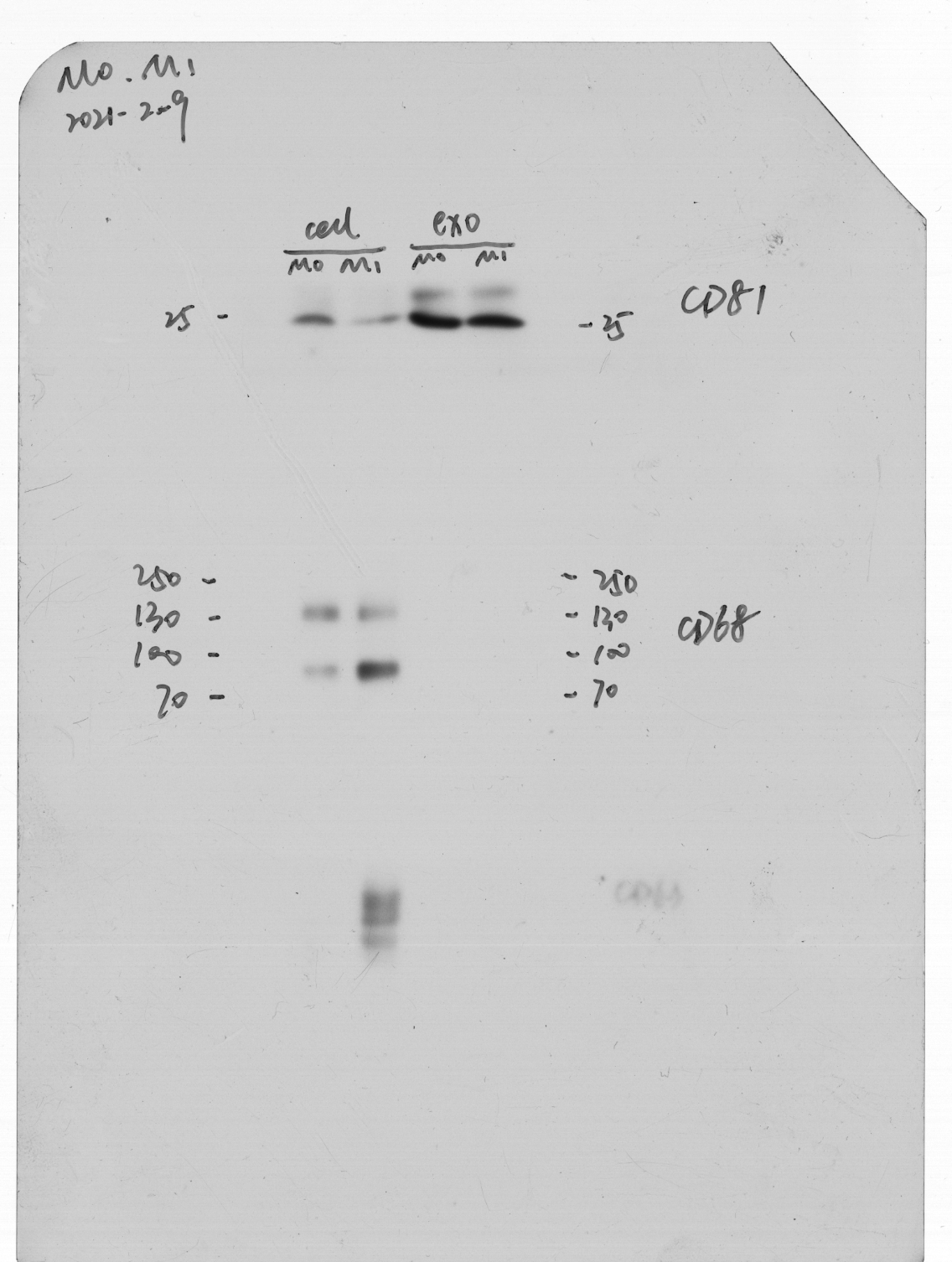
**

**Fig1C.CD81**


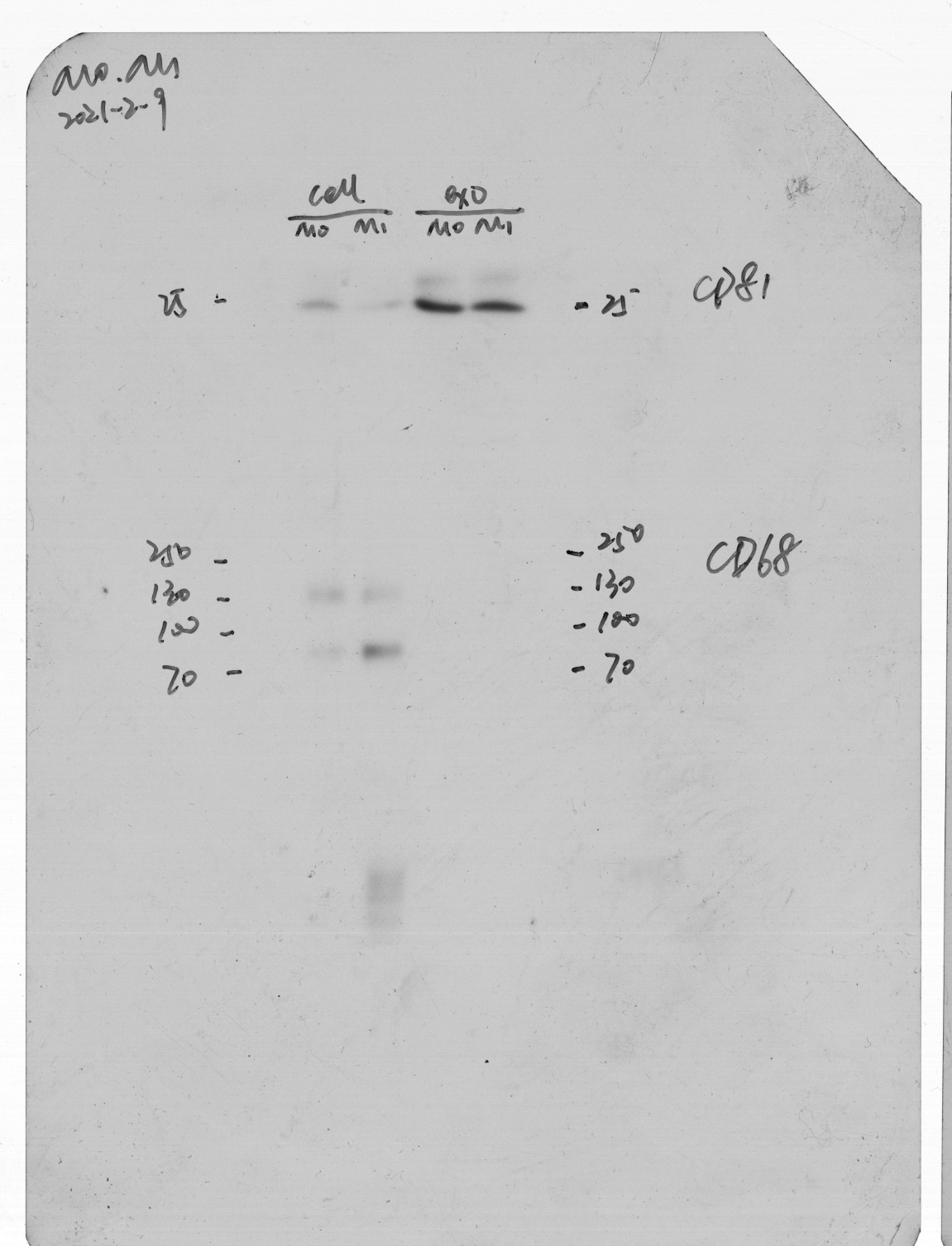


**Fig1C.CD63**


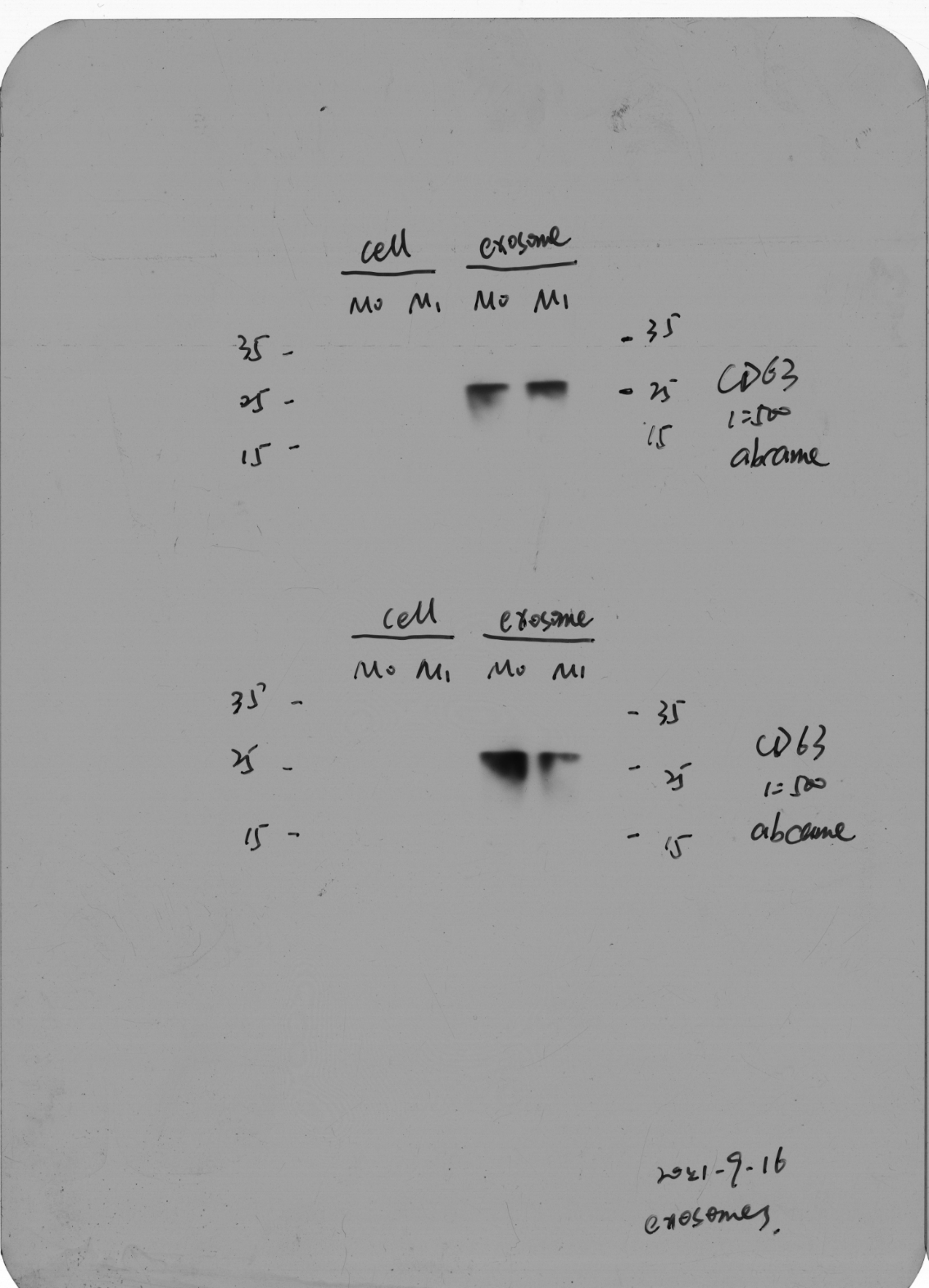


**Fig1C.GAPDH**

**
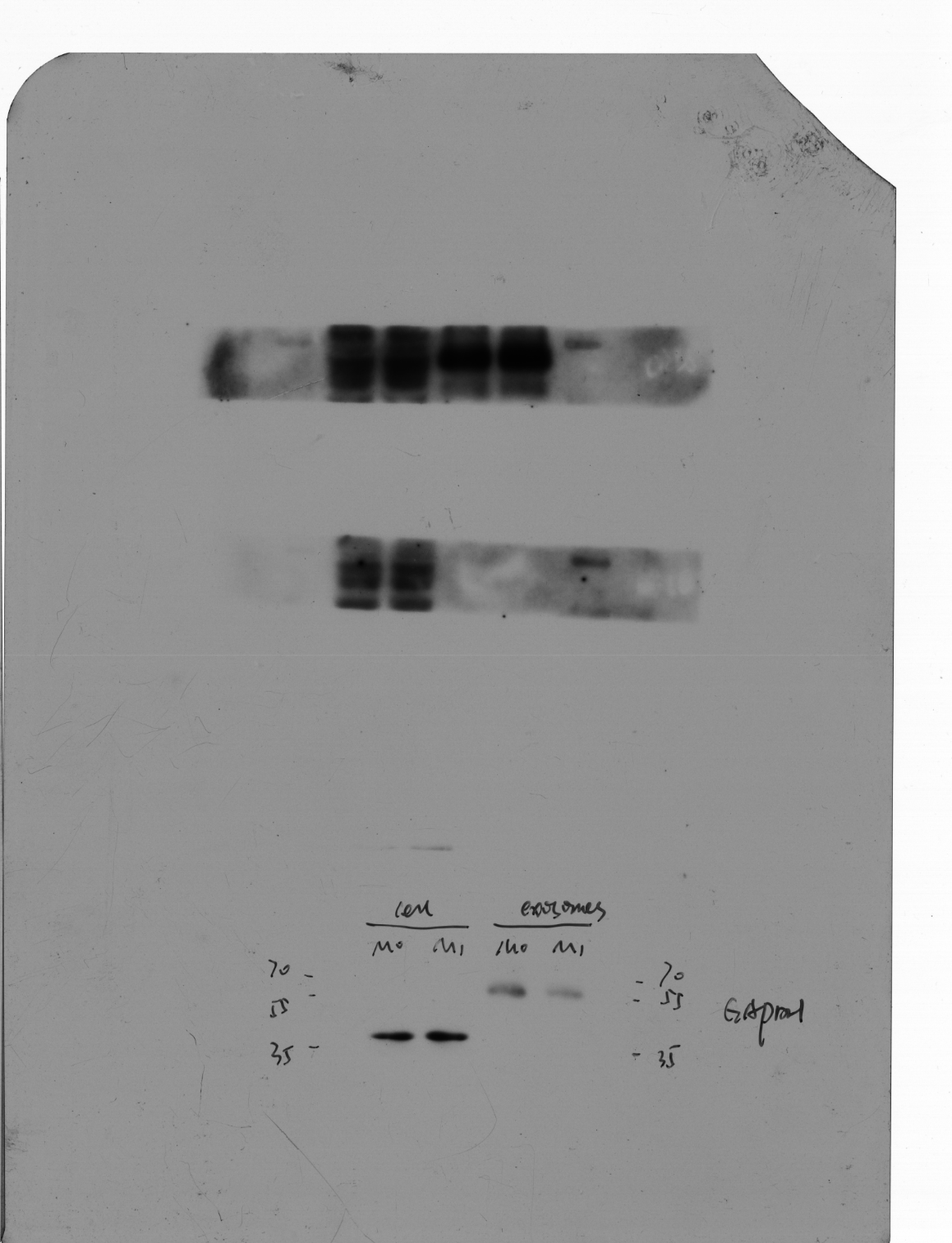
**

**Fig5D.IL-6R/GAPDH**

**
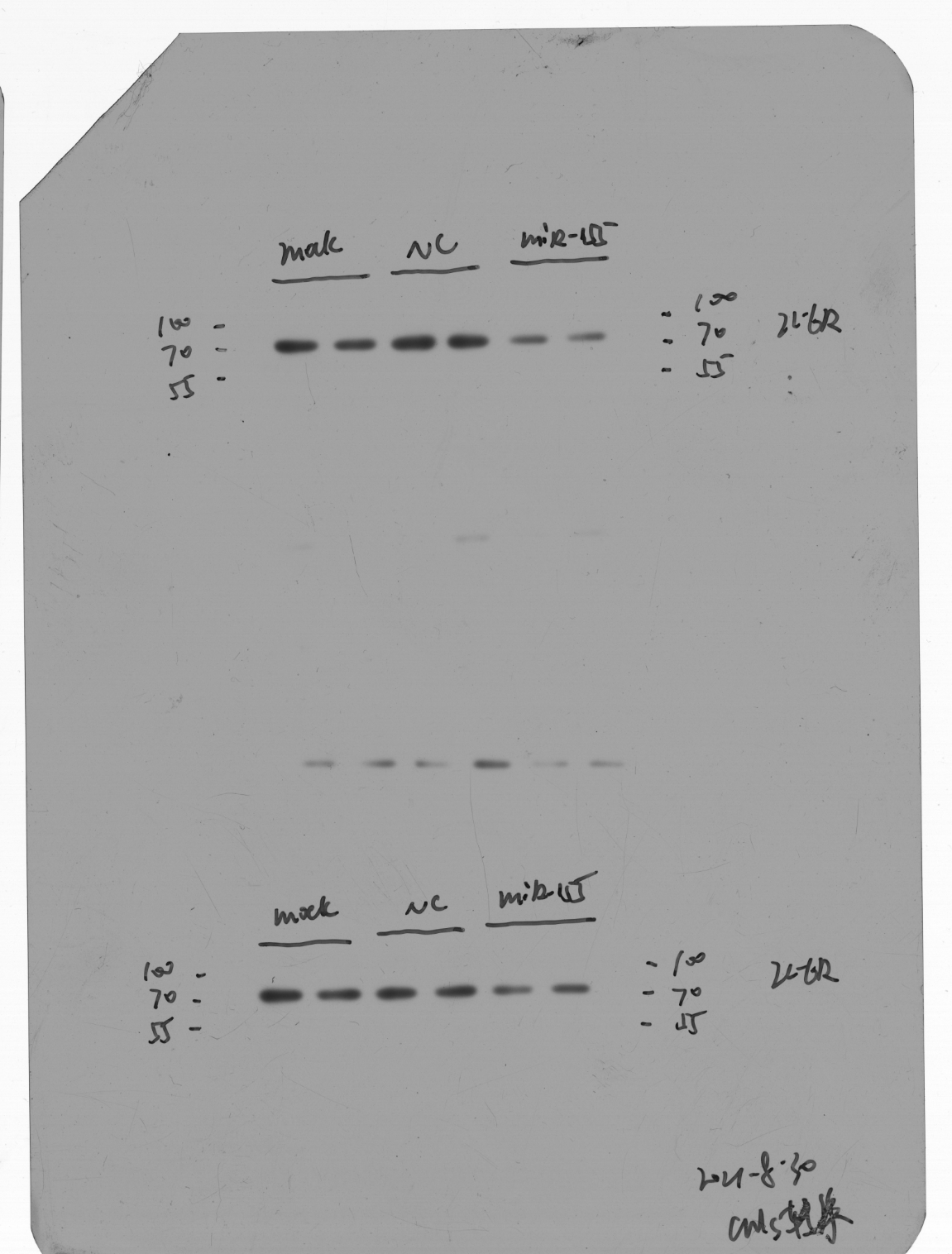
**

**Fig5D.GAPDH**

**
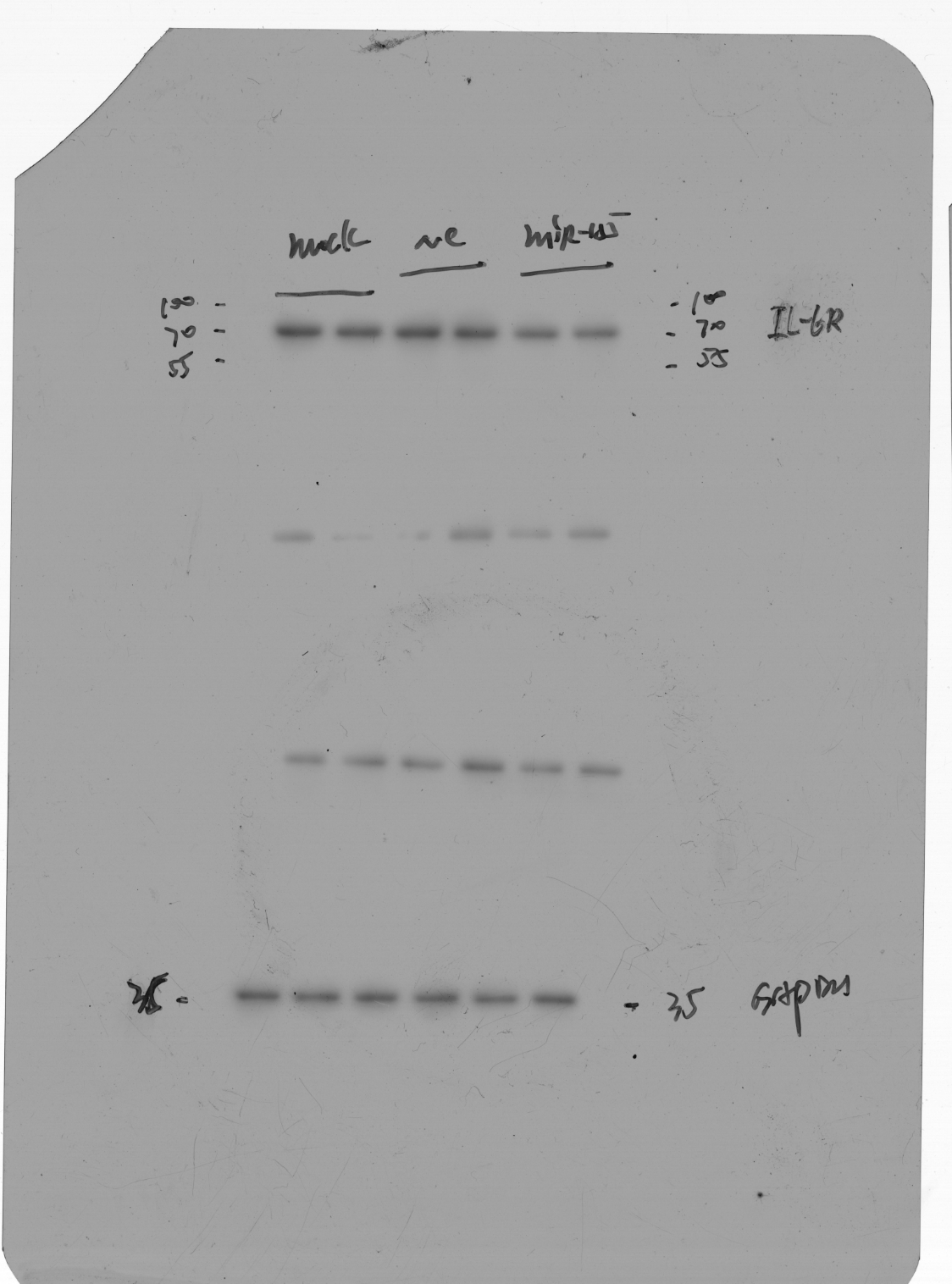
**

**Fig6C.IL-6R**


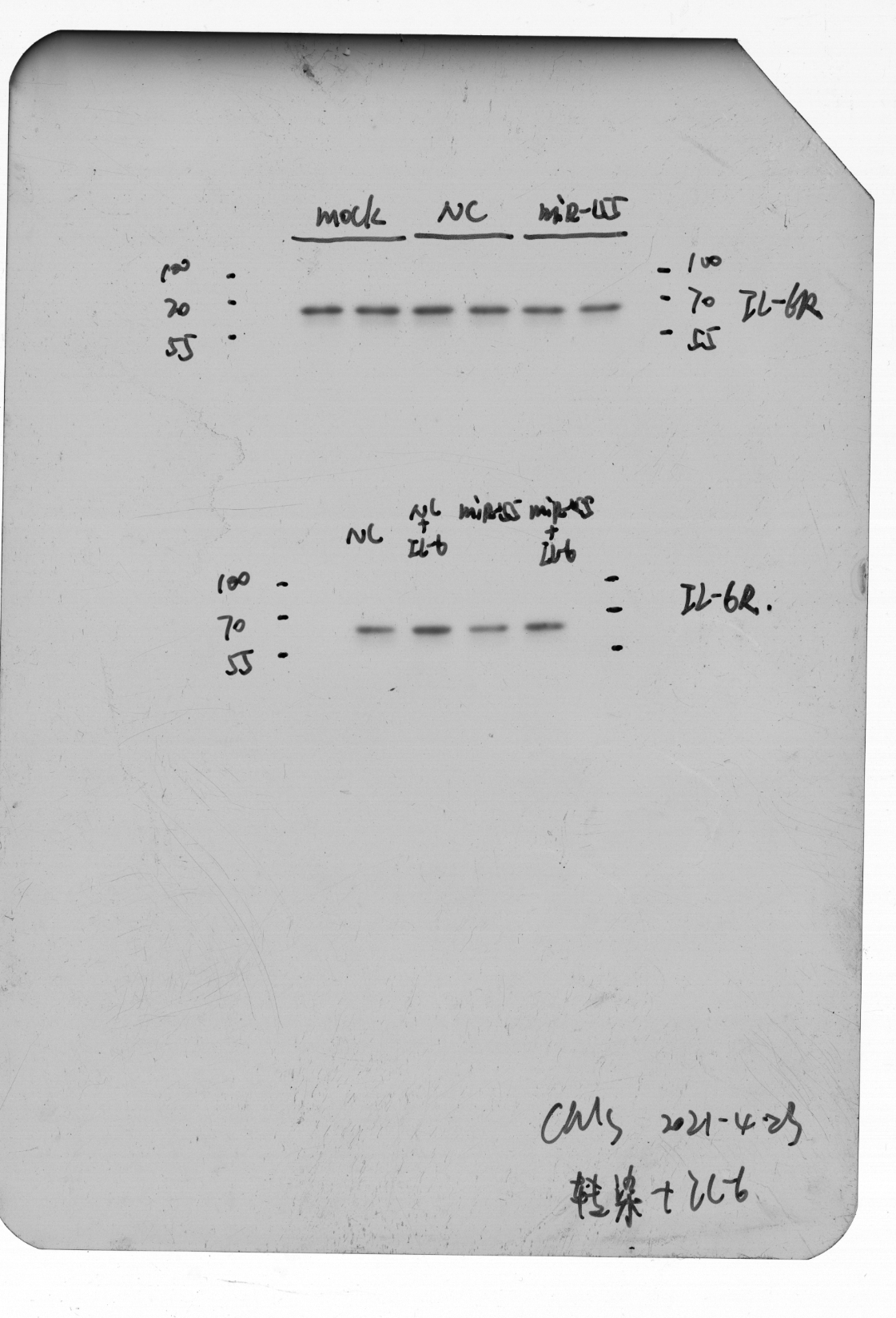


**Fig6C.p-JAK2**

**
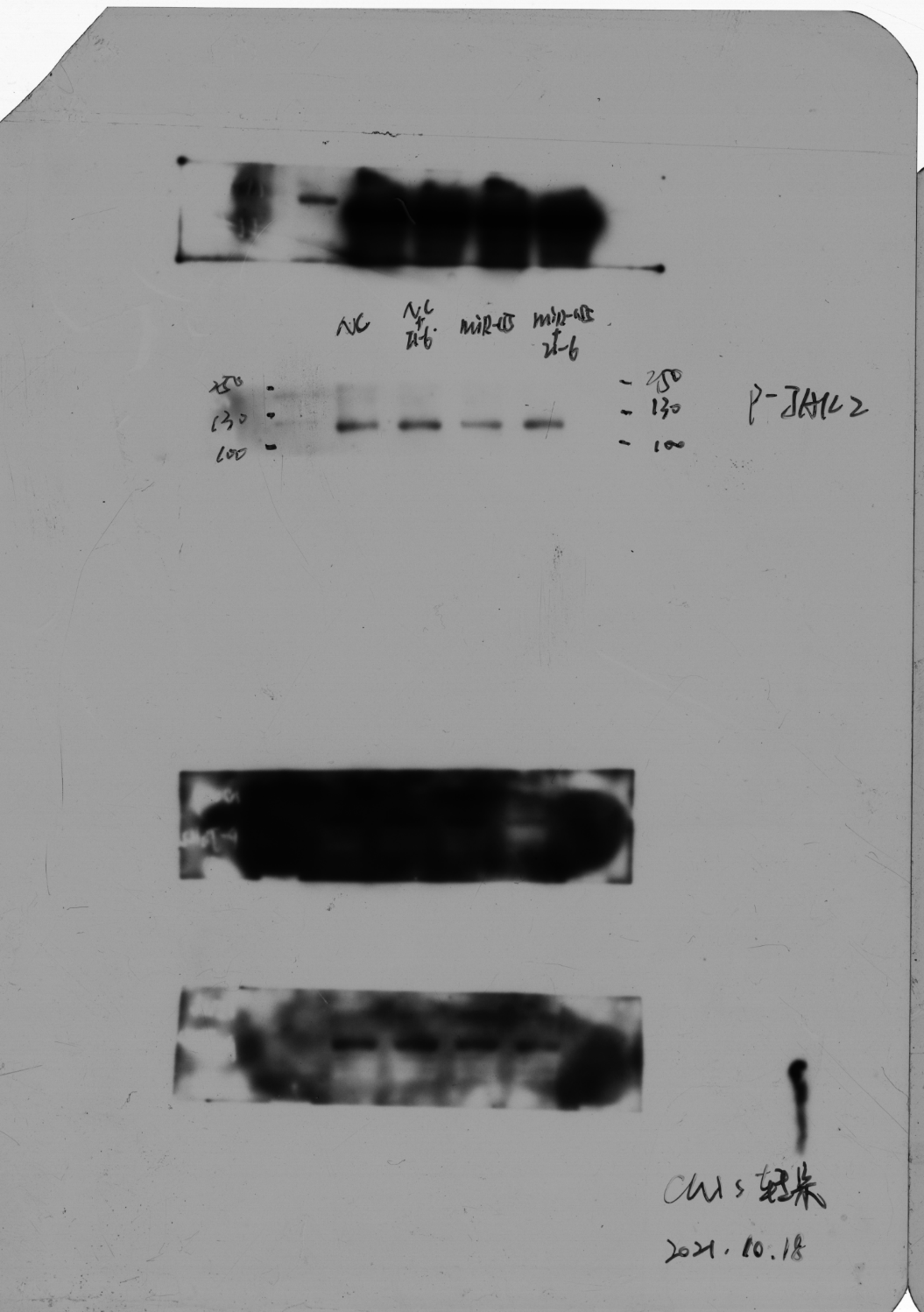
**

**Fig6C.p-STAT3**

**
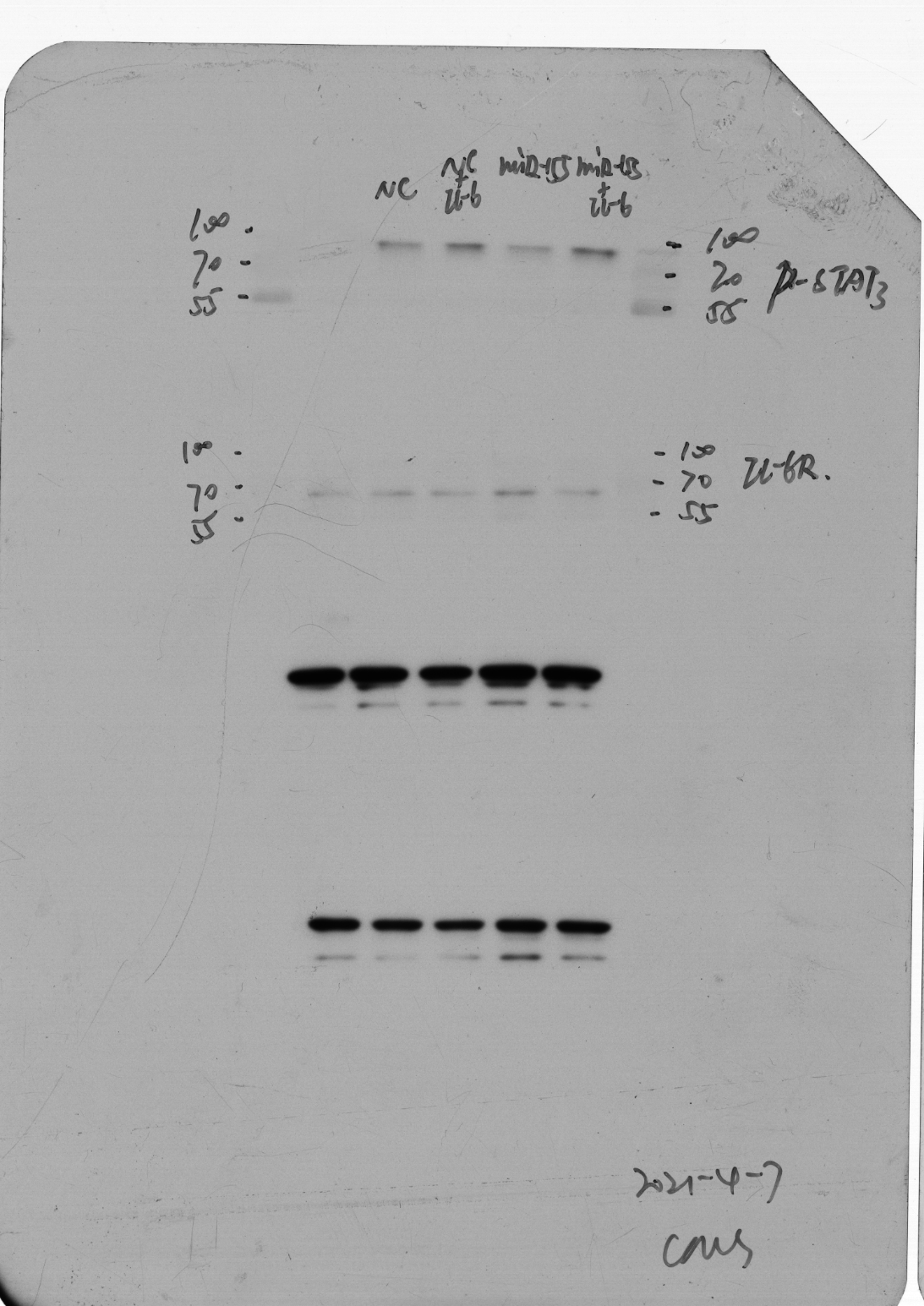
**

**Fig6C.GAPDH**


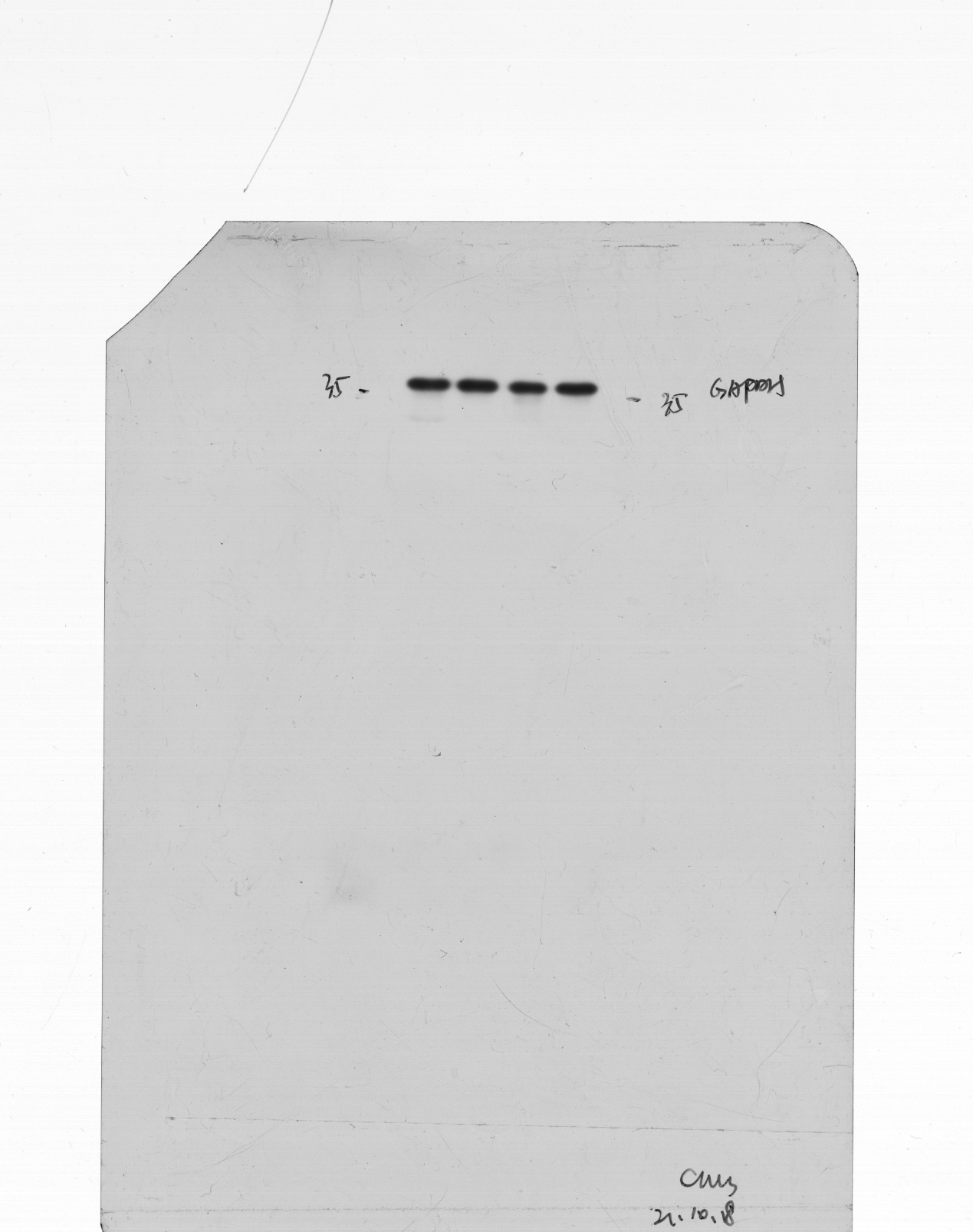

Supplement: Supplementary file 2 — Supplementary Material 2 [file 12872_2024_3893_MOESM2_ESM.docx]
